# Closing the mental health gap: transforming Pakistan's mental health landscape

**DOI:** 10.3389/frhs.2024.1471528

**Published:** 2025-02-05

**Authors:** Ambareen Main Thompson, Sheikh Mohd Saleem

**Affiliations:** ^1^ Primary Healthcare Specialist SINA Health Education & Welfare Trust, Karachi, Pakistan; ^2^ Independent Consultant and Public Health Researcher, Unicef India, New Delhi, India

**Keywords:** mental health, mental health crisis, multifaceted approach, multisectorial collaboration, primary health care, treatment gaps

## Introduction

Pakistan confronts a severe mental health crisis that compels urgent action. Mental disorders constitute a burgeoning global burden, with depression alone accounting for a staggering 4.4% of worldwide Disability-Adjusted Life Years (DALYs) (1). A stark inequity persists, with over 90% in low- and middle-income nations lacking access to mental health treatment, compared to over 50% receiving care in high-income countries (2). These disparities emanate from a chronic underinvestment, with low-income nations allocating a mere fraction, less than 1% of health budgets, to mental health (3).

Pakistan mirrors these global inequities. With a paucity of just 0.19 psychiatrists per 100,000 people (4), and an underwhelming allocation of only 0.4% of the health budget for mental health (5), Pakistan grapples to meet the needs of an estimated 24 million individuals requiring mental health services (6). Depressive, anxiety, and schizophrenia disorders are the most prevalent (7). Stigma surrounding mental illness remains an entrenched societal challenge (8).

Currently, Pakistan's mental health system operates primarily through tertiary care hospitals in major cities, with minimal integration into primary healthcare. Mental health services are largely concentrated in psychiatric departments of teaching hospitals, creating geographic and economic barriers for rural populations. The existing system relies heavily on psychiatrists and clinical psychologists, with limited involvement of general physicians, community health workers, or other non-specialist providers. Mental health education is notably absent from school curricula, and workplace mental health programs are virtually non-existent. Digital mental health solutions remain unexplored within the public sector, while community-based mental health services are severely limited. The proposed transformations would mark significant departures from this status quo through: task-sharing with non-specialist providers instead of exclusive specialist care; integration of services into primary healthcare facilities rather than tertiary hospitals alone; establishment of community clinics in place of centralized urban facilities; leveraging digital technology where traditional in-person care is the norm; and engaging community partners vs. the current isolated clinical approach.

**Figure 1:**
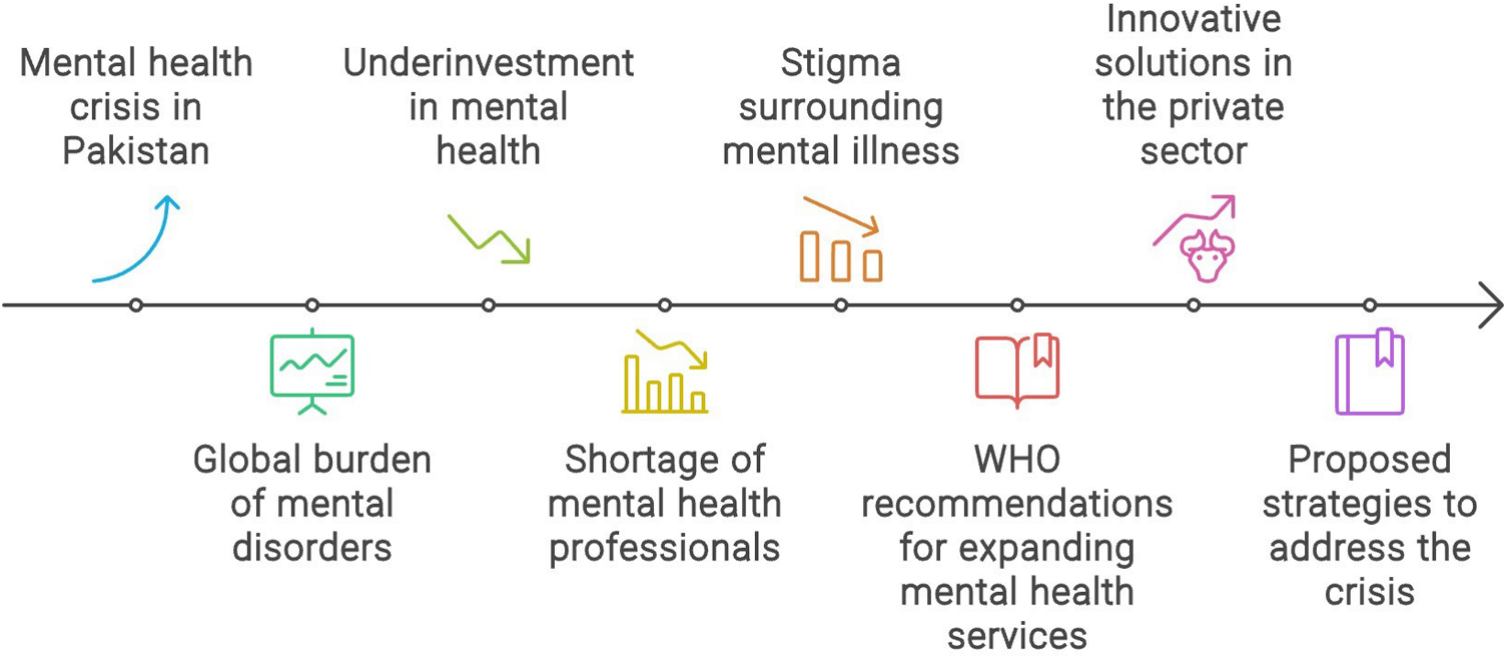
Factors accociated with mental health crisis in Pakistan.

To expand access, the WHO recommends strategies such as task-sharing care to non-specialist providers, integrating services into primary care and educational institutions, developing community clinics, leveraging digital technology, and engaging community partners (9). Pakistan could adapt approaches like training primary care workers in mental health protocols, building teacher capacity for school-based services, deploying lay counselors with specialist supervision, offering telemental health services, and engaging community health workers in outreach efforts (10) (Figure 1).

Amidst public sector constraints, Pakistan's private sector pioneers innovative solutions. NGOs like SINA- Health Education and Welfare trust conduct grassroots awareness campaigns, integrate mental health into community clinics, utilize telepsychiatry and digital tools, and address social determinants through campaigns on gender equity (11). Organizations like Pakistan Institute of Living and Learning (PILL) advocate for policies, build workforce capacity, and scale up culturally-adapted interventions (12). Digital startups like Sehat Kahani use telepsychiatry and mobile applications to bridge the workforce gap (13).

**Figure 2:**
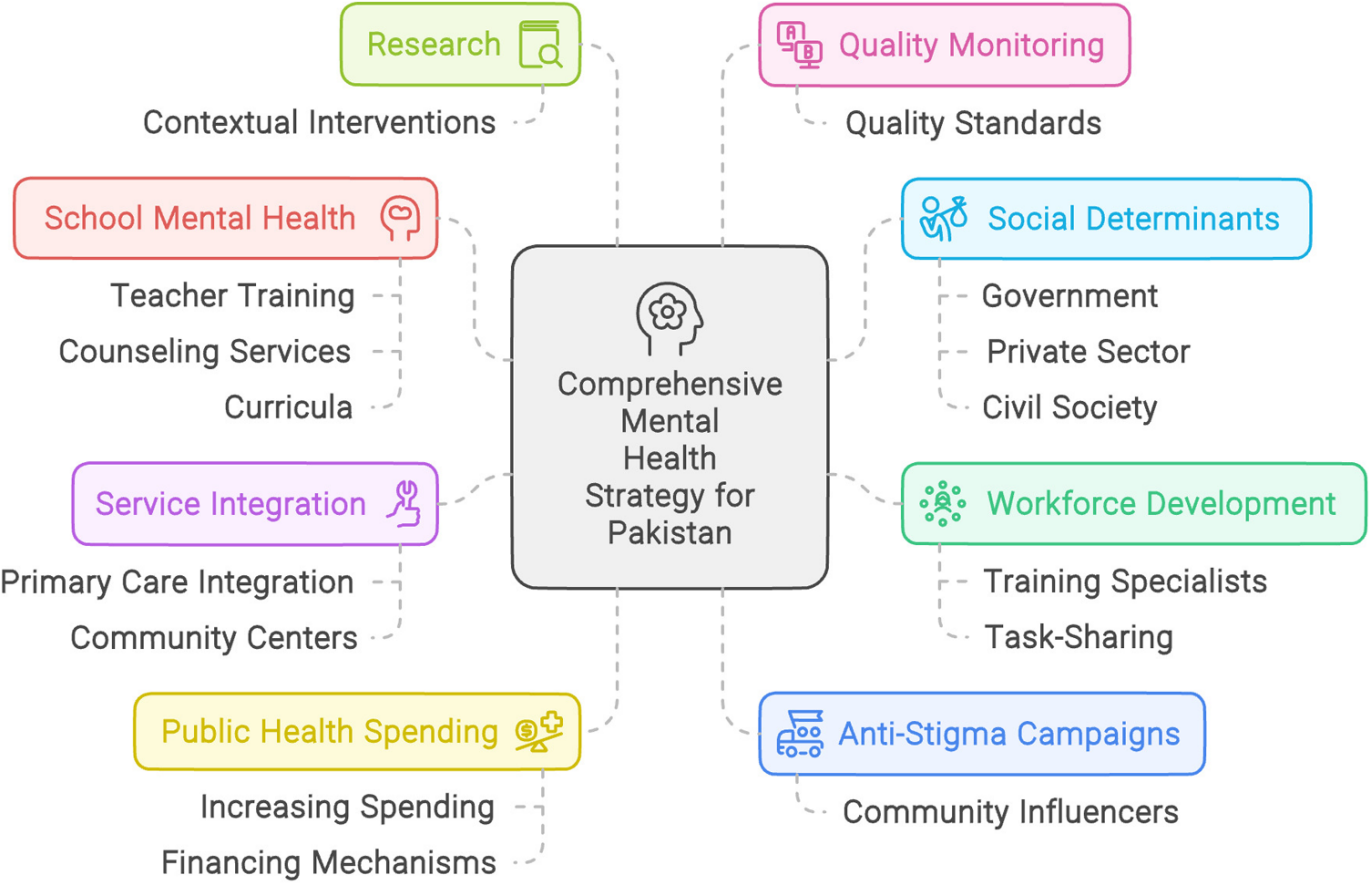
Mental health strategy for Pakistan.

A critical component in addressing Pakistan's mental health crisis is the implementation of comprehensive anti-stigma campaigns (14). These initiatives should operate at multiple levels based on established evidence (15) (Figure 2):

A. Community-level interventions (14, 15):
  (a) Engaging religious leaders and community elders to challenge traditional misconceptions
  (b) Training community health workers to provide accurate mental health information
  (c) Organizing community dialogue sessions and support groups
  (d) Using local media and art forms to share stories of recovery and hope
B. Educational initiatives (9, 16):
  (a) Incorporating mental health literacy into school curricula
  (b) Training teachers to recognize and respond to mental health concerns
  (c) Creating safe spaces for students to discuss mental health
  (d) Engaging parents through awareness programs
C. Media engagement (14, 15):
  (a) Partnering with media outlets to promote responsible reporting on mental health
  (b) Creating public service announcements featuring respected public figures
  (c) Using social media platforms to reach younger populations
  (d) Developing culturally sensitive content in local languages
D. Workplace programs (9, 16):
  (a) Implementing mental health awareness training in organizations
  (b) Establishing employee assistance programs
  (c) Creating supportive workplace policies
  (d) Reducing discrimination in employment practices

Research suggests such comprehensive anti-stigma campaigns can lead to (14, 15):

• Increased help-seeking behavior
• Earlier intervention and better outcomes
• Reduced discrimination
• Greater social support for individuals with mental health conditions
• Improved public understanding of mental health**

To comprehensively address the crisis, Pakistan must invest in scaling up its mental health workforce through training more specialists and task-sharing to non-specialists (3, 16). Integrating services into primary care and establishing community mental health centers is crucial for decentralizing access (9, 10). Increasing public mental health spending, developing sustainable financing mechanisms, and strengthening governance and policies are imperative (5, 16). Research on effective, contextually-appropriate interventions should guide investments (14). Developing quality monitoring mechanisms is key to ensuring standards of care (9).

Addressing social determinants through multi-sectoral coordination and whole-of-society approaches involving government, private sector, and civil society is vital (14). Sustained political commitment and strategic investments enabling universally accessible, community-based mental healthcare are crucial for realizing wellbeing for all Pakistanis (15, 16).

In essence, Pakistan confronts a formidable treatment gap with escalating rates of mental illness amid extreme limitations in mental health system capacity. Comprehensive strategies are necessitated, spanning workforce expansion, service integration into communities, increased financing, anti-stigma efforts, school interventions, research, quality assurance, and multi-sectoral coordination. While challenges are immense, prioritizing community-driven, decentralized mental health systems can ensure no individual is left behind on the path to greater wellbeing.
